# When gene flow really matters: gene flow in applied evolutionary biology

**DOI:** 10.1111/eva.12402

**Published:** 2016-07-16

**Authors:** Norman C. Ellstrand, Loren H. Rieseberg

**Affiliations:** ^1^ Department of Botany & Plant SciencesCenter for Conservation BiologyUniversity of CaliforniaRiversideCAUSA; ^2^ Botany DepartmentUniversity of British ColumbiaVancouverBCCanada

## Introduction

In the last half century, gene flow has moved from relative obscurity to a well‐recognized component of evolution. Gene flow, the successful transfer of alleles from one population to another, is now known to vary considerably among species, populations, and individuals as well as over time. It frequently occurs at rates sufficient to play an important evolutionary role for populations of both animals and plants (Ellstrand 2014; Yakimowski and Rieseberg 2014; Arnold 2015).

Gene flow does not automatically come to mind in the context of evolutionary applications. When the senior author shared the idea of a Special Issue on Gene Flow in Applied Evolution with a colleague, she asked, “What else is there besides crop breeding?” Considerably more, as we shall soon see.

Gene flow is important in a remarkable variety of applied situations. This Special Issue presents some representative points in the galaxy of applied topics in which gene flow plays a key role. The number of possible topics precludes an exhaustive treatment. Likewise, while we acknowledge that non‐sexual gene flow (horizontal transfer) has considerable applied and evolutionary significance in prokaryotes and eukaryotes (e.g. Koonin et al. 2001; Richardson and Palmer 2007; Arnold 2015), the topic is so large, we deem it worthy of separate treatment.

## When gene flow really matters

### Gene flow involving domesticated species

The recognition of gene flow's magnitude and variation has led to increased attention to gene flow in applied evolutionary biology. As noted above, breeders have long been acutely aware of gene flow. Intentional anthropogenic gene flow has long been practiced by breeders to deliver desirable traits to domesticated species via wide crosses followed by repeated backcrossing (e.g. Stalker 1980). But long before humans intentionally bred plants, spontaneous gene flow played a role in plant improvement. A few thousand years ago, spontaneous interspecific hybridization gave rise to first durum wheat and then bread wheat (Feldman et al. 1995). Likewise, lager beer yeast is the result of spontaneous interspecific hybridization and of a similar age (Hebly et al. 2015). Even natural hybridization within species has had its benefits. The evolution of highland maize (*Zea mays* ssp. m*ays*) landraces was the result of introgression from the wild *Z. m*. ssp. *mexicana* (Hufford et al. 2013).

But gene flow involving domesticated species is not universally beneficial. Pollen contamination from conspecifics and congeners growing within cross‐pollination distance can frustrate progress under artificial selection as well as jeopardize the genetic purity of seed multiplication programs (e.g. Kelly and George 1998). Spontaneous gene flow into or out of domesticated populations sometimes gives rise to new invasive and weedy lineages (Ellstrand et al. 2010). The rise of Africanized bees in the New World may be the best known example (Winston 1992). Another example is the case of the rise of herbicide‐resistant weedy rice in Brazil, reviewed in this issue (Merotto et al. 2016). A new herbicide‐resistant cultivar of cultivated rice had been introduced to facilitate the control of the conspecific weed. A local economic boom ensued as yields increased. But the combination of introgression of the resistance allele into the weed and strong selection by the herbicide lead to the dramatic evolutionary spread of weed populations bearing the allele for herbicide resistance, resulting in the reversal of economic fortunes. In extreme cases, the farmers were compelled to abandon their land.

The foregoing scenario involving a traditionally bred crop represents one of the primary concerns associated with the release of transgenic crops (Ellstrand 2001): that is, the delivery of transgenes into free‐living populations via gene flow might result in the evolution of new or more problematic weeds or invasives. It is not unusual for spontaneous gene flow from crops to their wild or weedy relatives to result in introgression (Ellstrand et al. 2013). But whether or not those alleles will persist and spread also depends on the relative fitness of free‐living plants that bear those alleles versus those that do not. Relevant multigenerational field experiments involving a variety of environments remain few.

One such study is presented in this Special Issue. Xia et al. (2016) measured the fitness effects of cultivated rice insect resistance transgenes that had been introgressed into various weedy rice lineages. The relative fitness effect of the transgene varied with both environment (level of insect infestation) and genotype (genetic background of the different lineages). Correspondingly, the Special Issue includes a review of field studies of the fitness effects of crop transgenes with wild or weedy ancestors (Lu et al. 2016). The review reveals that the relative fitness of lineages that are the result of gene flow between a transgenic crop and wild/weedy relative cannot easily be predicted *a priori*. The presence of transgenes sometimes correlates with increased relative fitness, decreased relative fitness, or no change in relative fitness. Likewise, enough studies have accumulated to demonstrate that genetic background and environment can sometimes play a key role in determining the direction and magnitude of transgene fitness effects.

Two decades after the commercial release of genetically engineered crops, gene flow has enabled crop transgenes to move into and apparently persist in free‐living populations. The known cases are relatively few and mostly involve feral canola (Ellstrand 2012). Thus far, the economic and environmental impacts of transgenes in free‐living populations have been nil or, at worst, mild. As noted above, gene flow between traditionally improved domesticates and their wild relatives sometimes results in lineages that are not so benign.

### Gene flow and the evolution of novel weeds and invasives

The evolution of novel weeds and invasives need not involve domesticates at all. Natural gene flow and subsequent admixture involving only non‐domesticated taxa are known to have preceded the evolution of dozens of new weedy and invasive lineages (Schierenbeck and Ellstrand 2009). Examples include plants, animals, and microorganisms. Recently, an increasing number of studies have revealed that even within‐species admixture appears to have played a role in the evolution of many invasive lineages (Rius and Darling 2014). In that case, admixture depends on multiple introductions (Bock et al. 2015). Likewise, a small, but growing, number of examples of invasives that evolved from intertaxon hybridization have been shown to be the result of multiple, independent hybridization events.

One such example is in this Special Issue. The tumbleweed, *Salsola ryanii,* evolved in California as recently as a few decades ago and is certainly no more than a century old. An allohexaploid derivative of hybridization between *Salsola australis* and *Salsola tragus,* its known range expanded from a few isolated populations in California's Central Valley to a broad distribution in that Valley as well as California's coastal valleys in roughly a decade. Despite its recent origin and rapid spread, molecular analysis by Welles and Ellstrand (2016) demonstrates that this neo‐invasive is the result of three independent admixture events and that gene flow among the individual lineages is just beginning, leading to within‐species admixture.

### Gene flow and conservation

The rise of conservation genetics in the 1980s started with a focus on fragmentation and small populations, featuring the hazards associated with drift, inbreeding, and lack of variation in the face of environmental challenges (Schonewald‐Cox et al. 1983). Early on, gene flow was seen largely as a benefit for populations at risk of extinction as an agent that would reverse those hazards (Lacy 1987).

The intentional introduction of new genetic diversity for the purposes of sustaining a population is called “genetic rescue” (Tallmon et al. 2004). For small, inbred populations, outcrossing with conspecific populations with similar environmental adaptations typically does lead to an increase in fitness. Indeed, in a recent meta‐analysis, positive fitness effects were report in 93% of such cases (Frankham 2015). A caveat is that Frankham (2015) focused on systems that are at low risk for outbreeding depression, and his conclusions may not apply to cases where gene flow occurs between genetically and/or ecologically divergent populations.

Of course, the addition of genetic diversity almost always includes the introduction of new individuals. Thus, genetic and demographic relief can be confounded. In this Special Issue, Fitzpatrick et al. (2016) estimate the relative contributions of genetic and demographic factors to the rescue of two wild guppy populations from Trinidad. Using molecular markers to distinguish between native and immigrant genotypes, and to determine the parentage of offspring, they were able to show that the demographic contribution of the new immigrants was indeed substantial. However, hybrid genotypes were major contributors to population expansion as well, possibly due to heterosis. Thus, both genetic and demographic factors play an important role in the evolutionary rescue of these populations.

As conservation genetics began to mature, gene flow was no longer perceived solely as a panacea for the ills of small populations. Significant genomic or eco‐genetic differences between source and recipient populations can result in a fitness drop in interpopulation hybrids, often deemed “outbreeding depression.” If differences are primarily genomic, the fitness drop will tend to be environment independent. If the differences are eco‐genetic, immigrant gene flow will disrupt local adaptation (Price and Waser 1979; Edmands 2007).

Gene flow from common species has put rare species at increased risk of extinction by genetic swamping, where the local genotypes are replaced by hybrids, or by demographic swamping, where population growth rates are reduced due to outbreeding depression. In this issue, Todesco et al. (2016) conduct a literature survey to identify and rank factors affecting extinction risk through hybridization. They report that the risk of extinction through hybridization is strongly linked to human activities such as the intentional release of captive‐bred individuals, introductions of non‐native taxa, and habitat disturbance, whereas strong reproductive barriers between hybridizing taxa greatly reduces risk. Hybridization asymmetry was found to matter as well, with extinction risk increasing when the rare taxon acts as the maternal parent. This finding makes sense because females typically invest more resources in offspring production than males. Interestingly, genetic rescue was rare in the case studies included in the literature survey, which mostly involved interspecific gene flow, and genetic swamping was much more frequently reported than demographic swamping.

Hybridization involving rare populations also has important practical implications. Should the hybrids be protected for the sake of preserving important pools of genetic diversity or should they be destroyed to slow the process of genetic assimilation? Regardless of the answer to these questions, it is critical that we have robust methods for detecting hybrids (Payseur and Rieseberg 2016) and making inferences about different histories of hybridization. Here, Gompert and Buerkle (2016) demonstrate using computer simulations that while it is possible to distinguish between some scenarios of hybridization and selection, false signals of hybridization can arise as well because of limited geographic sampling. They also show that hybrids are highly variable in phenotype and fitness, both within and between hybrid classes. They argue that such variability will limit our ability to predict the outcome(s) of hybridization in individual cases and thereby hinder the development of management plans.

### Gene flow and policy

Gene flow is now the subject of study beyond the field and the laboratory. When appropriate, decision‐makers are incorporating information and questions about gene flow in policy. Ridley and Alexander's (2016) article in this Special Issue examine the work of two United States Environmental Protection Agency (EPA) Science Advisory Panels, a 2000 panel on genetically engineered crops and a 2009 panel on watershed connectivity, as examples in which the extension of gene flow science informs the development of policy. They also reveal that it is the role of certain “boundary organizations”, such as the EPA, to engage in a type of “intellectual gene flow” to successfully deliver information accumulated in one field (evolutionary biology) to one in which that information is applied. Clearly, if gene flow science is now a source of information that contributes to policy, the 21st century is “When Gene Flow Really Matters”.
